# Prevalence of polypharmacy among older adults in Ethiopia: a systematic review and meta-analysis

**DOI:** 10.1038/s41598-023-45095-2

**Published:** 2023-10-17

**Authors:** Tegene Atamenta kitaw, Ribka Nigatu Haile

**Affiliations:** Department of Nursing, College of Health Science, Woldia University, Woldia, Ethiopia

**Keywords:** Health care, Geriatrics

## Abstract

Polypharmacy is a significant concern for older adults. Taking multiple medicines to prevent and treat comorbidities is very common in older adults, potentially leading to polypharmacy. Polypharmacy is associated with the development of geriatric syndromes, including cognitive impairment, delirium, falls, frailty, urinary incontinence, and weight loss. The prevalence of polypharmacy varies according to the literature. There is a paucity of data regarding the prevalence of polypharmacy among older adults. Therefore, this study aimed to estimate the pooled prevalence of polypharmacy among older adults in Ethiopia. A comprehensive search of databases, including PubMed, MEDLINE, EMBASE, Hinari, Cumulative Index to Nursing and Allied Health Literature, International Scientific Indexing, Cochrane library and Web of Science, and Google Scholar, was conducted. STATA statistical software (version 17) was used to analyze the data. Forest plot and I^2^ heterogeneity test were computed to examine the existence of heterogeneity. Subgroup analysis and sensitivity analysis were done to explore the source of heterogeneity. Publication bias was evaluated by using funnel plots and Egger’s test. A random effect model was used to determine the pooled prevalence of polypharmacy. After reviewing 123 studies, 13 studies with a total of 3547 older adults fulfilled the inclusion criteria and were included in this meta-analysis. The result from 13 studies revealed that the pooled prevalence of polypharmacy among older adults in Ethiopia was 37.10% (95CI: 28.28–45.91). A Subgroup Meta-analysis showed that the heterogeneity level was slightly lower among studies done in Oromia region (I2 = 46.62, P-value = 0.154). Higher pooled polypharmacy prevalence was found among older adults with cardiovascular disorders (42.7%) and admitted patients (51.4%). In general, it was found that the pooled prevalence of polypharmacy among older adults in Ethiopia was high. More than one in three older adults take five or more medications at a time. Thus, intervention focusing on rational geriatric pharmacotherapy is significant to prevent unnecessary pill burden, adverse drug events, medical costs, geriatric morbidity, and mortality. Furthermore, enhancing pharmacist roles towards medication therapy management and safety monitoring in older adults is also indicated.

## Introduction

Taking medications for chronic diseases to alleviate symptoms and prevent disease progression is common among older adults. However, taking too many medications might be harmful. Drug-related harm among older adults is one of the most challenging public health issues globally^1^. Polypharmacy was formerly used to refer to issues related to multiple drug consumption and excessive or unnecessary drug use^2,3^.

Even though the actual definition of “polypharmacy” is varied in some literature, the widely accepted definition by most literature and the WHO (World Health Organization) is taking five or more medications daily^4–9^.

Polypharmacy is a significant concern for older adults. Older people are more susceptible to adverse drug reactions (ADRs) due to age-related changes in the metabolic system and a decrease in drug clearance, further exacerbating the increase in drug usage^10^.

The more drugs, the higher the risk of drug interactions. According to the studies, people who take five to nine drugs have a 50% probability of experiencing adverse drug reactions^11^. The pharmacokinetics, pharmacodynamics, comorbidity, and medication regimens are all significantly affected by ageing, which may raise the likelihood of adverse drug reactions^12^. Age-related altered drug response decreased renal and hepatic function, hypoalbuminemia, lower body weight, and multi-morbidity, which may raise the risk of polypharmacy and further contribute to adverse drug reactions^13^. The most common chronic conditions leading to polypharmacy among older adults are cardiovascular disease, hypertension and diabetes^14^.

The likelihood of having multiple chronic illnesses rises with increased aging. Consequently, the prevalence of having two or more diseases reaches approximately 40% among older adults, which may rise as age increases. Taking multiple medicines to prevent and treat comorbidities is very common in older adults, making them vulnerable to polypharmacy^15^.

Polypharmacy has significant consequences for older adult health. It is associated with developing geriatric syndromes, including cognitive impairment, delirium, falls, frailty, urinary incontinence, and weight loss^16^. Medication-related problems associated with polypharmacy were common in older adults, such as inappropriate prescribing, poor adherence, over dosage, under dosage, inappropriate drug selection, inadequate monitoring, adverse drug effects, and drug interactions^17^. Polypharmacy and medication-related problems among older adults are associated with increased healthcare costs, hospital admissions, and adverse health consequences, including falls, cognitive impairment, and reduced quality of life^18^. 30% of an increase in medical costs is attributable to polypharmacy due to increases in outpatient visits, hospitalizations, and potentially inappropriate medication^19^. Hyper polypharmacy (10 or more drugs) is also common in older adults, with a prevalence of 5.1%^20^.

The prevalence of polypharmacy varies according to the literature. It ranges between 4% and 96.5% depending on age group, definition of polypharmacy, and healthcare setting^7,21^. The prevalence of polypharmacy increases with age. Overall, the prevalence was 25.3% in people aged 65–74 years, 36.4% in those aged 75–85 years, and 46.5% in those over 85 years old. The prevalence of polypharmacy does not significantly differ between males and females^22^.

Studies among older adults from different countries showed different rates of prevalence. In Sweden 45% among age above 75 years^23^, in Australia 36% among age above 70 year^24^, Belgium(8%)^25^, China(55.4%)^26^, (India 25.2%)^27^, Saudi(51.5%)^28^ and Nigeria (23.8%)^29^.

In Ethiopia, single-area studies show that the prevalence of polypharmacy ranges from 10.8% to 64.3%^30,31^. Cardiovascular disease, diabetes, comorbidity, and being overweight are among the risk factors for polypharmacy in older adults^32,33^. The prevalence of potentially inappropriate medication (PIM) related to polypharmacy is also high (47.2%)^34^. Besides, 75% of older adults in Ethiopia have poor quality of life due to polypharmacy^35^. In Ethiopia, little effort has been made so far to reduce the consequences of polypharmacy by reducing irrational medication prescriptions. Some interventions included introducing a ward-based clinical pharmacy service and establishing standard treatment guidelines monitored by the Ethiopian Food and Drug Authority^36,37^.

It is essential to determine how frequently these high-risk drugs are prescribed to the older population, especially to individuals who are already on multiple medicines. In addition to effectively treating those chronic illnesses, encouraging wise drug prescribing for older patients may be necessary to reduce adverse drug events (ADEs) and unintended pill burden. The prevalence of polypharmacy varies among different countries and settings. There is inconclusiveness in polypharmacy prevalence reports in different regions. Thus, this study aimed to determine the pooled prevalence of polypharmacy among older adults in Ethiopia.

## Methods and analysis

### Protocol development

This protocol was designed in accordance with preferred reporting Standards for Systematic Reviews and Meta-Analysis Protocols^38^. This review will be conducted in accordance with the recommended methods of meta-analysis in observational Studies^39^. The study protocol was registered in PROSPERO (CRD42022364456).

### Search strategy

A compressive literature search was conducted using different databases. Databases that were included: MEDLINE, EMBASE, Hinari, Cumulative Index to Nursing and Allied Health Literature, International Scientific Indexing (ISI), Cochrane Database of Systematic Reviews and Web of Science (WoS) and Google Scholar. Searching was conducted using alternative keywords such as prevalence, magnitude, burden, epidemiology, polypharmacy, and Ethiopia. For instance, the search techniques in PubMed appeared as follows: (((((Polypharmacy [MeSH Terms]) OR (polytherapy [Title/Abstract])) OR (polyprescription [Title/Abstract])) OR (Multiple medications [Title/Abstract])) OR (multidrug therapy [Title/Abstract])) OR (multiple pharmacotherapies [Title/Abstract])) AND (Ethiopia [Title/Abstract]). Grey literature and manual search were also attempted to retrieve unindexed research articles. The results were limited to studies conducted in humans and available in the English language only. Snowballing technique was used to ensure the comprehensiveness of search and to identify relevant primary studies. The search activity was done by TA and RN.

### Study eligibility

#### Inclusion criteria

The inclusion criteria was determined based on the CoCoPop mnemonic—Condition, Context, and Population (Table 1), following the recommendation for reviewing prevalence and/or incidence studies^40^.

**Table 1:** Inclusion criteria based on the CoCoPop mnemonic principle.

Condition	Outcome of interest should measure the prevalence of polypharmacy
Context	Setting should be in regions within Ethiopia
	All observational and population-based studies reporting prevalence of polypharmacy
Population	Older adults ≥ 65 year
	Patients with polypharmacy

#### Exclusion criteria

The review exclude the following studies: (1) Studies that did not report the prevalence of polypharmacy in Ethiopian older adults; (2) narrative reviews, expert opinions, case reports, duplicate studies, and interventional studies; (3) Articles with no complete text and data that is difficult to extract after contacting the corresponding author(s) was excluded.

### Data extraction and management

Two reviewers (TAK and RNH) conducted data extraction independently using a standardized extraction form. After a systemic search was conducted in different databases, potentially eligible articles based on the inclusion criteria were imported to EndNote 20. Potential duplicates were considered and removed if two or more citations shared the same author, title, publication date, volume, issue, and start page information. The studies were screened and selected for full-text review based on the inclusion criteria. Data abstraction was done using structured forms prepared in Microsoft Excel spreadsheet format. The extracted data form includes study identifiers, the primary investigator’s name, study region, year of publication, sample size, setting, study design, and admission status. In addition, the prevalence of polypharmacy was extracted from each study using the number of participants declared to have polypharmacy as the numerator and the total number of sample sizes as the denominator. The corresponding author was contacted when any difficulties were encountered during data extraction.

### Risk of bias (quality) assessment

The JBI (Joanna Briggs Institute) critical appraisal tool was used to assess the quality of the included studies. The tool contains questions that are evaluated individually and replied with “Yes,” “No,” or “Unclear”. Quality analysis of the studies was conducted by two independent reviewers. Disputes were discussed and resolved by consensus among the authors and an independent reviewer. MOOSE statement was used to report the result of systematic review and meta-analysis^41^. The score is 1 for “Yes”, 0 for “no”, and U for “unclear”. The final Scores for each study were summed and transformed into a percentage. Finally, ranking was given as follows: ≤ 49% = high risk of bias, 50–69% = Moderate risk of bias, and above 70% = low risk of bias. Only studies that scored ≥ 50% were considered in this systemic review and meta-analysis. In the case of ongoing disputes between reviewers, the average ratings of the reviewers were computed. The quality of the primary study results was recorded in a separate column in the data extraction form.

### Statistical analysis

The extracted data were exported to Stata 17 software for analysis. Pooled prevalence was reported as percentages with a 95% CI. A random-effects model was employed. Forest plot and I^2^ statistics heterogeneity test were computed to examine the existence of heterogeneity. I^2^ statistics result is interpreted as follows: ≥ 75% = considerable, 50–75% = substantial, 25%–50% = moderate and ≤ 25% = low heterogeneity^42^. The existence of heterogeneity is declared if I-squared (I^2^) result is ≥ 50% and the p-value is less than 0.05. Thus, further subgroup and sensitivity analyses were computed to explore the source of heterogeneity. Publication bias was evaluated for the prevalence of polypharmacy by using funnel plots and Egger’s test. Subgroup analysis was done using study region, publication year and sample size, study quality, setting, and study population characteristics.

### Ethical approval

A formal ethical statement is not required because this systemic review used publicly accessible data and did not disclose the article's authors by name.

## Results

### Searching results

**Figure 1:**
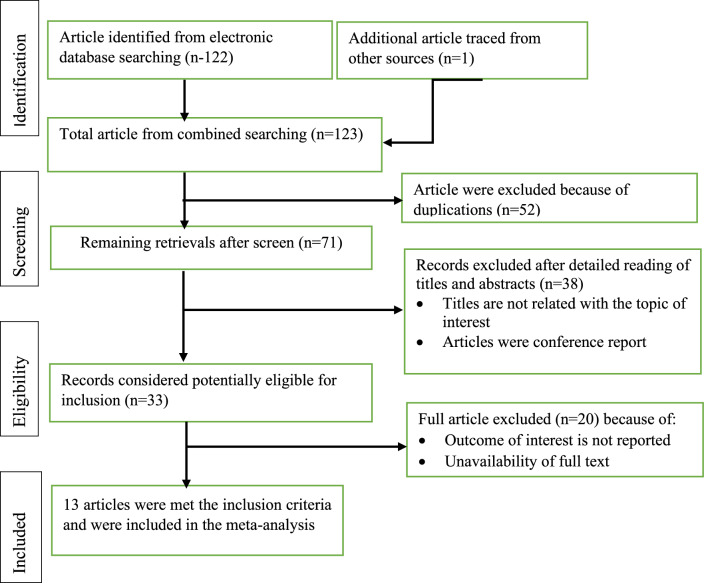
Flow chart diagram describing selection of studies for the systematic review and meta-analysis of prevalence of polypharmacy among older adults in Ethiopia, 2022.

A total of 123 records were retrieved from different database search engines. 52 of them were excluded because of duplications through the EndNote citation manager. From 71 records, 38 retrievals were excluded because they were inappropriate to the outcome of interest in this meta-analysis. The remaining 33 records were potentially eligible for inclusion. After thoroughly checking the full publications of 33 articles, 20 studies were removed because their outcome estimates varied from the outcome of interest. Finally, 13 eligible studies were included in this systematic review and meta-analysis to estimate the pooled prevalence of polypharmacy among older adults in Ethiopia (Fig. 1).

### Characteristics of original studies

As indicated in Table 1, all studies employed cross-sectional study design. The small and large sample sizes were recorded in the studies conducted by Geresu et al. (116) and Lemma et al. (400), respectively. The highest prevalence (64.3%) of polypharmacy among older adults was reported from the study conducted by Dagnew et al., and the lowest proportion (10.8%) was reported from a study done by Lemma et al. All of these studies were conducted from the year 2016 to 2022. In this meta-analysis, a total of 3547 older adults were included to estimate the pooled prevalence of polypharmacy. From the eleven regions and two-city administrations within the country, three regions and one city administration were included in this meta-analysis. Five studies from Amhara region, three studies from Oromia region, one study from Tigray region, and four studies from Addis Ababa city. There have been no reports from the remaining regions so far (Table 2).

**Table 2:** Characteristics of original studies on prevalence of polypharmacy among older adults in Ethiopia, 2022.

Author	Publication year	Region	Study area	Study design	Sample size	Prevalence (%)	quality score
Tefera et al.^33^	2020	Amhara	Gondar	Cross-sectional	153	31.4	7
Tegegn et al.^43^	2018	Amhara	Gondar	Cross-sectional	351	17.7	8
Assefa et al.^32^	2020	Addis Ababa	Addis Ababa	Cross-sectional	255	42.7	7
Adem a Tegegne^44^	2022	Addis Ababa	Addis Ababa	Cross-sectional	384	53.1	8
Tesfaye et al.^45^	2021	Oromia	Jimma	Cross-sectional	219	42.5	7
Geresu et al.^46^	2017	Oromia	Shashemene	Cross sectional	116	32.8	6
Teka et al.^47^	2016	Tigray	Mek'ele	Cross sectional	140	57.9	8
Dagnew et al.^30^	2022	Amhara	Northwest Ethiopia	Cross sectional	389	64.3	9
Lemma et al.^31^	2020	Addis Ababa	Addis Ababa	Cross sectional	400	10.8	9
Hailu et al.^48^	2020	Oromia	Jimma	Cross sectional	200	35.5	8
Solomon^49^	2021	Addis Ababa	Addis Ababa	Cross sectional	376	47.9	7
Sada^50^	2017	Amhara	Dessie	Cross-sectional	244	23.0	7
Bhagavathula et al.^34^	2021	Amhara	Gondar	Cross-sectional	320	24.1	8

### Quality of the included studies

All studies were assessed using the JBI (Joanna Briggs Institute) critical appraisal tool for prevalence studies. The result of the JBI assessment tool revealed that all of the studies had a six or above score, giving the lowest percentage of 66.7% (Table 2)***.***

### Prevalence of polypharmacy (meta-analysis)

**Figure 2:**
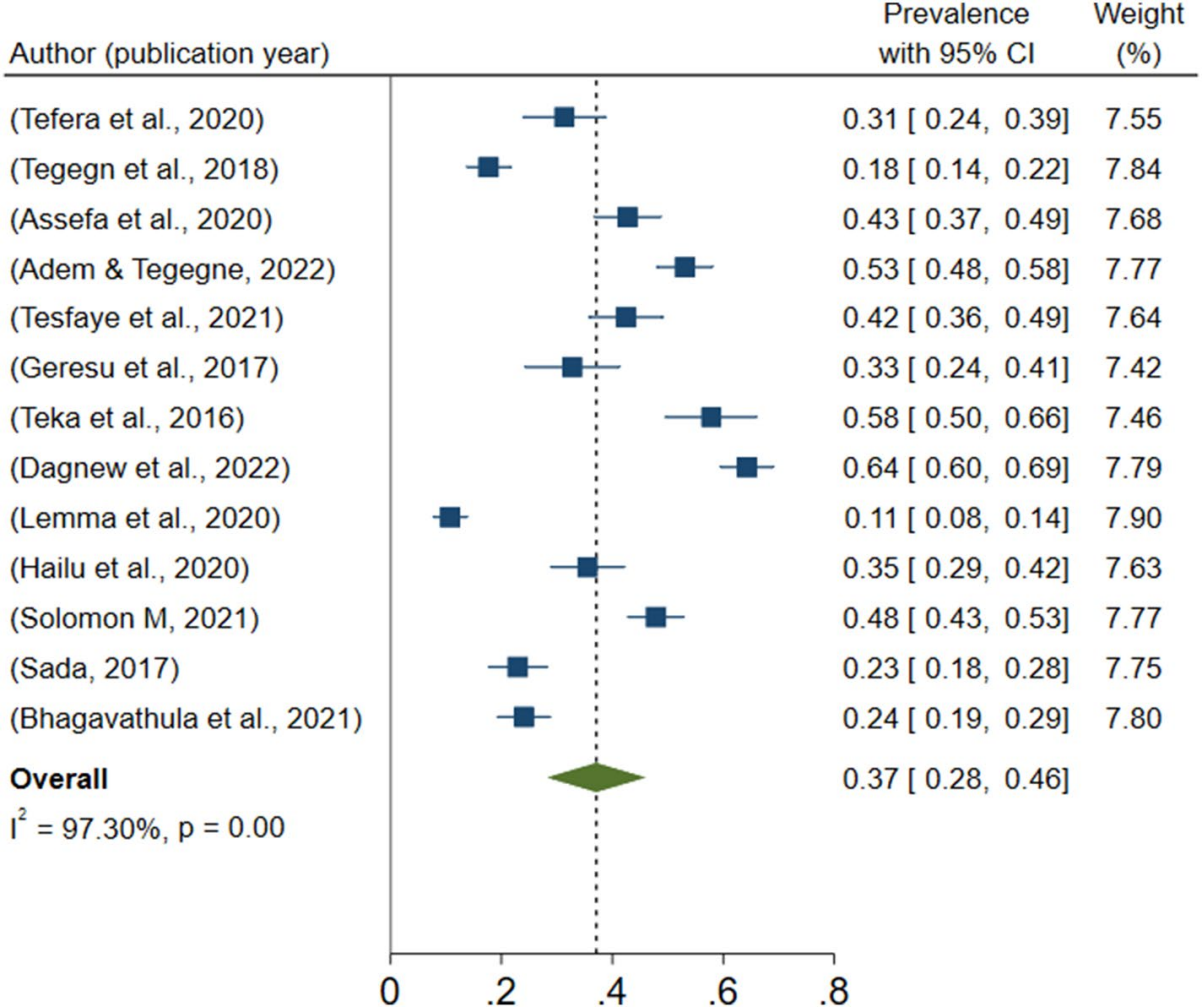
The pooled prevalence of polypharmacy among older adults in Ethiopia, 2022.

This meta-analysis identified considerable heterogeneity across the studies (I2 = 97.3%, P-value = 0.000). As a result, we used a random effect model to estimate the pooled prevalence of polypharmacy. The results of 13 studies revealed that the pooled prevalence of polypharmacy among older adults in Ethiopia was 37.10% (95 CI: 28.28–45.91) (Fig. 2).

### Publication bias

Substantial publication bias was assessed objectively by using both Begg’s and Egger’s tests. The result of both Begg’s and Egger’s tests revealed that there is no publication bias at p-values of 0.6693 and 0.1922, respectively.

### Subgroup analysis

This meta-analysis exhibited considerable heterogeneity. Thus, to identify the possible sources of heterogeneity, subgroup analysis was done using study region, publication year, sample size, study quality, setting, and study population characteristics.

The subgroup analysis indicated that the heterogeneity level was slightly reduced among studies done in Oromia (I2 = 46.62, P-value = 0.154). Subgroup analysis by study region/city showed that the highest estimated prevalence found in studies conducted in Addis Ababa city was 38.5% (95 CI: 19.72, 57.35), and the lowest prevalence was found in Amhara regional state 32.1% (95 CI: 15.63, 48.49). By publication year, the pooled estimate of polypharmacy prevalence was higher among articles published between 2019 and 2022 than among articles published before 2018 (39.1% vs. 32.6%). There was considerable heterogeneity within both groups. By median sample size (≤ 250 vs. > 250), there is no significant difference in pooled prevalence between groups (37.0% vs. 37.1%). Based on the JBI score, the pooled prevalence estimates among studies scoring above and below 80% were 37.5% and 36.8%, respectively. There was also considerable heterogeneity within both groups. The highest pooled polypharmacy prevalence was obtained among studies done in inpatient settings, 51.4% (95 CI: 39.07, 63.74). Furthermore, studies conducted among cardiovascular patients counted the highest pooled prevalence among other patients (42.7% vs. 35.5%) (Table 3).

**Table 3:** Subgroup analysis of polypharmacy pooled prevalence estimation in Ethiopia, 2022.

Moderator variables	Variable category	Included studies	Prevalence % (95% CI)	I^2^%	% P-value
Study region/city	Amhara	4	32.1 (15.63, 48.49)	98.15	0.000
	Oromia	4	37.3 (31.63, 42.97)	46.62	0.154
	Addis Ababa city	3	38.5 (19.72, 57.35)	98.51	0.000
Publication year	2018 and before	4	32.6 (15.16, 49.97)	97.00	0.000
	2019–2022	9	39.1 (28.55, 49.65)	97.43	0.000
Sample size	≤ 250	6	37.0 (27.48, 46.51)	91.19	0.000
	> 250	7	37.1 (22.34, 51.96)	98.71	0.000
JBI score (%)	≤ 80%	6	36.8 (29.15, 44.42)	88.74	0.000
	> 80%	7	37.5 (21.83, 53.15)	98.72	0.000
Setting	Inpatient	4	51.4 (39.07, 63.74)	94.23	0.000
	Outpatient	6	29.8 (17.05, 42.52	97.76	0.000
	Both	3	32.7 (21.17, 44.31)	89.52	0.000
Patient character tics	Cardiovascular patients (Heart Failure)	3	42.7 (30.37, 54.92)	91.84	0.000
	Other	10	35.5 (24.58, 46.41)	97.84	0.000

### Leave-one-out meta-analysis

Leave-one-out analysis was conducted to explore the influence of a single study on the overall effect size estimate. Leave-one-out meta-analysis omits the corresponding study and performs a meta-analysis on the remaining set of studies (n−1 studies). If the cross-ponding study confidence interval doesn’t include the overall effect size estimate (theta), it is declared that the study has a significant influence on the overall effect size estimate^51^. In this study, the overall effect size estimate (theta) is 0.371 and is included within the confidence interval of all studies. Thus, omitting one study does not significantly influence the overall effect size estimate (Table 4).

**Table 4:** Leave one out meta-analysis to explore the influence one study on the overall pooled prevalence estimation in Ethiopia, 2022.

Omitted study	Effect size	95% CI	P-value
(Tefera et al., 2020)	0.376	(0.281, 0.471)	0.000
(Tegegn et al., 2018)	0.387	(0.298, 0.477)	0.000
(Assefa et al., 2020)	0.366	(0.271, 0.462)	0.000
(Adem & Tegegne, 2022)	0.357	(0.266, 0.449)	0.000
(Tesfaye et al., 2021)	0.367	(0.271, 0.462)	0.000
(Geresu et al., 2017)	0.375	(0.279, 0.470)	0.000
(Teka et al., 2016)	0.354	(0.265, 0.443)	0.000
(Dagnew et al., 2022)	0.348	(0.265, 0.430)	0.000
(Lemma et al., 2020)	0.393	(0.310, 0.477)	0.000
(Hailu et al., 2020)	0.372	(0.277, 0.468)	0.000
(Solomon M, 2021)	0.362	(0.268, 0.456)	0.000
(Sada, 2017)	0.383	(0.290, 0.475)	0.000
(Bhagavathula et al., 2021)	0.382	0.289, 0.475)	0.000
Theta	**0.371**	**(0.283, 0.459)**	**0.000**

Significant values are in [bold].

## Discussion

The prevalence of polypharmacy in geriatric patients has increased over time, highlighting an urgent need to develop new strategies for managing the high medication burden in the older population. This systematic review and meta-analysis aims to assess the pooled prevalence of polypharmacy among older adults in Ethiopia. The findings from 13 studies indicated that the pooled prevalence of polypharmacy among older adults in Ethiopia is 37.1%. More than one older person in three takes more than five drugs at a time. This finding is lower than the study done in highly populated countries such as China (48%)^52^ and India (49%)^53^. This difference might be attributable to China and India being the top two countries with the largest number of older adults. Older adults with non-communicable diseases and multimorbidity also increase in those countries^54^. This tends to increase the number of prescribed medicines at the same time.

The finding of this study is higher than the study done in developed countries such as New Zealand (29.5%)^55^, USA (9%)^56^ and Belgium (8%). However, nearly similar to this study, there is a considerable high prevalence of polypharmacy reported in a study done in developing countries 33%^57^. The possible explanation for the high prevalence of polypharmacy in developing countries might be due to non-adherence to standard treatment guidelines. Furthermore, it is common to prescribe more than one medication to target a single disease due to a lack of a specific drug and a need for more sophisticated laboratory testing to identify a causative agent. Approximately 29% of hospitalized geriatric patients have drug side effects^58^. This is attributable to a high prevalence of polypharmacy. Older patients suffer from side effects of drugs because of the increased number of prescription medicines for unidentified diseases and the constant change in the drug list.

The subgroup analysis of this study showed that the prevalence of polypharmacy was higher among older adults with cardiovascular disease (42.7%). This finding is in a row with previous studies that report high polypharmacy prevalence in cardiovascular patients^59^ and^60^. The trend of non-cardiovascular comorbidities among hospitalized heart failure patients has been rising over time^61^ and is linked to adverse outcomes and an increased burden of non-CVD medicines. The extent of multimorbidities in the cardiovascular patient was high, and non-cardiovascular medications significantly contributed to the patient's level of polypharmacy^25^. Since older adults with cardiovascular disease are at risk for an increase in the number of medications they use at a time, prescriptions, including choice medications for the cardiovascular patient, should be a precaution to prevent adverse drug reactions.

In addition, the polypharmacy prevalence was higher among hospitalized older patients than ambulatory patients (51.4% vs. 29.8%). Similarly, a study conducted among older adults in Australia also reported a high prevalence of polypharmacy in admitted patients (52.2%). Patients admitted to the hospital have more severe and complicated cases requiring aggressive management. Thus, prescribers tend to try multiple and different medications at a time.

The limitation of this study was that only articles written in English were included in the analysis. Besides, the studies included in this meta-analysis were from three regions and one city administration. Thus, the finding might luck a national representative. Furthermore, Substantial heterogeneity within the included studies was detected.

## Conclusion

Keeping older people healthy is a significant concern, considering the number of people over 60 years globally, which is expected to double by 2050 (to 2.1 billion)^62^, and has already become a valuable resource for all of society. Therefore, this meta-analysis was crucial to understand the level of medication utilization among geriatrics who require long-term clinical care and are at risk for medicine-related unfavorable outcomes. The result of this meta-analysis suggested high-level polypharmacy among the older population. More than one in three older adults is subjected to polypharmacy. Thus, National drug policy should encourage and enhance rational and efficient uses of medicine to prevent adverse outcomes such as increased healthcare costs and the deterioration of older population health conditions due to significant drug-drug interactions. Furthermore, a high prevalence of polypharmacy is detected among patients with cardiovascular disorders and admitted patients. Therefore, clinicians should stick with the treatment guidelines and ensure the relevance of the pharmaceutical target medicine to prevent unnecessary pills from being burned in those groups. In addition, further research studies regarding prescribing practice, adherence to treatment guidelines, and inappropriate polypharmacy among the older population are highly recommended.

## Data Availability

Data will be available upon request of the corresponding author.
